# A Comprehensive sLORETA Study on the Contribution of Cortical Somatomotor Regions to Motor Imagery

**DOI:** 10.3390/brainsci9120372

**Published:** 2019-12-13

**Authors:** Mustafa Yazici, Mustafa Ulutas, Mukadder Okuyan

**Affiliations:** 1 Department of Computer Engineering, Faculty of Engineering, Karadeniz Technical University, 61080 Trabzon, Turkey; ulutas@ktu.edu.tr; 2 Department of Physiology, Faculty of Medicine, Karadeniz Technical University, 61080 Trabzon, Turkey; mokuyan@ktu.edu.tr

**Keywords:** EEG, motor imagery, somatomotor area, brain–computer interface (BCI)

## Abstract

Brain–computer interface (BCI) is a technology used to convert brain signals to control external devices. Researchers have designed and built many interfaces and applications in the last couple of decades. BCI is used for prevention, detection, diagnosis, rehabilitation, and restoration in healthcare. EEG signals are analyzed in this paper to help paralyzed people in rehabilitation. The electroencephalogram (EEG) signals recorded from five healthy subjects are used in this study. The sensor level EEG signals are converted to source signals using the inverse problem solution. Then, the cortical sources are calculated using sLORETA methods at nine regions marked by a neurophysiologist. The features are extracted from cortical sources by using the common spatial pattern (CSP) method and classified by a support vector machine (SVM). Both the sensor and the computed cortical signals corresponding to motor imagery of the hand and foot are used to train the SVM algorithm. Then, the signals outside the training set are used to test the classification performance of the classifier. The 0.1–30 Hz and mu rhythm band-pass filtered activity is also analyzed for the EEG signals. The classification performance and recognition of the imagery improved up to 100% under some conditions for the cortical level. The cortical source signals at the regions contributing to motor commands are investigated and used to improve the classification of motor imagery.

## 1. Introduction

The brain is one of the most complex organs in the body, as it controls our biological functions and mental tasks simultaneously. Advances in medicine, physiology, engineering, and neuroimaging have helped scientists understand the brain. In the last couple of decades, it has been shown that external devices can be controlled via EEG signals acquired by sensors, by using signal processing and classification algorithms. In the literature, the brain–machine interface, neural interface, neural prosthetics, and neural engineering terms are also used for brain–computer interfaces (BCIs). Researchers have shown that a person can control cursor movement on a screen or control a robotic or prosthetic arm or leg by using brain signals. The design and implementation of BCI is a multidisciplinary field in which physicians, psychologists, and engineers work together to develop better BCIs. Engineers use advanced signal processing techniques to both filter and extract features and machine learning methods for better signal analysis. A large number of BCI applications have been developed recently, and there has been an increasing amount of interest in this area [1]. Research on how the brain stores information and processes both stored and sensory inputs to generate motor functions has contributed a vast amount of knowledge on the matter, thanks to the availability of low cost and high-performance hardware, as well as open-source software. Engineers use machine learning methods to better learn from a limited number of features and accurately classify signals.

**Figure 1:**
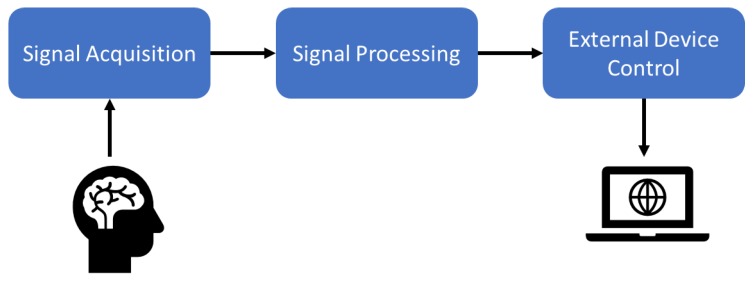
A brain–computer interface (BCI) system.

Researchers have proposed advanced signal amplifiers, filters, A/D converters, recording devices, and embedded computers to run new machine learning and artificial intelligence algorithms. A general BCI scheme is given in Figure 1.

There are some restrictions on using BCIs in daily life. Even though a BCIs’ EEG signal classification accuracy cannot reach 100%, several methods have been proposed to improve the accuracy and reliability of BCIs. BCIs are not easy to use, as patients do not feel comfortable. Moreover, sometimes BCIs can be dangerous due to misclassified signals. BCIs are still not permitted in daily life, except in research centers where they are tested in a controlled environment. The number of successful applications must increase for manufacturers to meet the demand for cost-effective BCIs. The proposed method aims to improve the classification accuracy for non-invasive BCIs. There are also invasive and semi-invasive BCI systems, but non-invasive EEG signal acquisition remains better for patients’ health and relaxation. One of the aims of EEG studies is to obtain information without using expensive radiodiagnosis techniques, which are not included in this study. Better success can be achieved with the support of radiodiagnosis data, such as fMRI or MEG, which we plan to use for future work.

BCI technology is used to control prosthetic devices by transforming motor imagery thoughts into motor functions to help paralyzed patients and amputees. It is important for a successful BCI to interpret precisely what the subject imagines by analyzing his or her brain waves. BCI techniques are used for control, assessment, and rehabilitation. Thus, both engineers and neuroscientists are working together to interpret thoughts by using brain signal analysis.

Motor imagery signals have been classified using various methods in the literature. Murguialday [2] used 8–13 Hz (μ) band activity for BCI control in 2013. Yuan [3] reviewed sensorimotor rhythms on BCIs in 2014. Handiru et al. [4] studied right and left-hand motor imagery on cortical levels in 2015. Ang and Guan [5] studied the control and rehabilitation potential of BCIs on stroke patients in 2017. Xia et al. [6] moved an on-screen cursor in two dimensions in 2017. In 2018, Li et al. [7] studied motor imagery tasks with the same dataset used in this work and showed that source domain analysis outperforms sensor domain analysis. Alazrai et al. [8] reported success in finger movements, Lu et al. [9] controlled a vehicle using EEG signals, and Xygonakis et al. [10], in 2018, studied four-class motor imagery in the EEG source space and improved its accuracy compared to the sensor data analysis. Qingsong et al. [11] analyzed four-task motor imagery in 2019, while Zhang et al. [12] reported in 2019 that children can successfully use BCIs.

In this work, both the acquired sensor and computed source space signals are used for improved classification of motor imagery using EEG signal analysis. EEG signals are first transformed to sources using inverse problem solution methods. The obtained cortical signals and sensor EEG signals are used together for improved classification. It is shown that augmenting the sensor signals via somatomotor region sources yields better results than those of similar studies reported in the literature. Our aim is to develop a BCI for paralyzed and disabled people, to transform their motor imagery thought signals to motor signals to control external devices. The selected somatomotor area signals improve classification success, as shown in the results. Successful BCIs will soon be able to be designed by interpreting not only the sensor but also the source signals. Furthermore, the known motor signal generating regions of the brain are tested for the improved classification of motor imagery.

Since most of the cortical studies in the literature achieved better results than the sensor level studies, we used cortical level signals in this work. Moreover, the sLORETA approach, which yields superior accuracy performance than the other methods, was selected to map the EEG signals to the cortical ROIs. Our aim is to find the most relevant ROIs for the right hand and right foot motor imagery in order to develop a BCI to transform motor imagery signals to control external devices. The main novelty of our study to improve the accuracy of the right hand and right foot imagery classification problem using only nine ROIs, excluding those not relevant to the chosen motor imagery tasks (i.e., the primary lip motor area (M1L), somatosensory association cortex (SAC), and secondary somatosensory area (S2)). We analyzed the recorded EEG signals using a 10–5 system with 118 channels. We used more channels, as suggested by many researchers, to achieve a better inverse solution.

## 2. Materials and Methods

### 2.1. BCI Competition Dataset

The BCI competition dataset IVa for the BCI competition III is used in this study [13]. There are recordings of five healthy subjects in this dataset. The subjects sat in a comfortable armchair and performed the imagery tasks shown by the cues. Subjects were given three motor imagery duties, (L) left hand, (R) right hand, and (F) right foot. Visual cues were indicated for 3.5 s. The EEG signals were sampled at 1000 Hz and then down-sampled to 100 Hz. Only the right hand and right foot signals were used for competition. Many training and test sets were made, each with a different number of samples. One of the aims in the competition was to achieve successful classification with the test sets while using small training sets. Two-second motor imagery time slice recordings are also used in this work. There were a total of 280 training and test sets and 118 channels used for recording the EEG data. The number of training and test trials are listed in Table 1 for each subject in the dataset.

**Figure 2:**
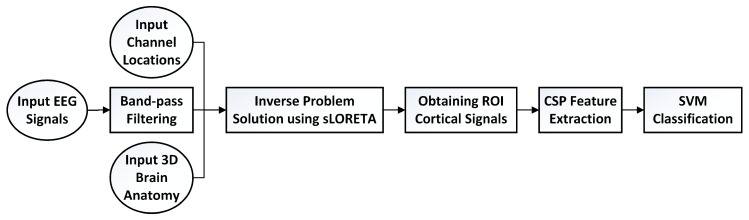
Flow diagram of this work.

Flow diagram of this work is given in Figure 2.

### 2.2. Preprocessing

Because motor imagery is one of the important ERPs (event-related potentials), the EEG signals are sampled at 100 Hz. The motor-related μ rhythms at 8 to 13 Hz are filtered from the raw EEG signals by a band-pass filter. Signals in the 0.1–30 Hz delta, theta, alpha, and beta rhythms and 8–13 Hz μ rhythms are used to compare and determine the most relevant regions. In the results section, the results for both 0.1–30 Hz and the μ band analysis are given.

#### 2.2.1. Head Model

We assumed head tissue conductivity values of 1 S/m for both the scalp and brain and 0.0125 S/m for the skull, as suggested by Liu et al. [14] for the layer conductivity values.

#### 2.2.2. Number of Electrodes and Locations

The coordinates of the EEG measurement electrodes must be known to determine the potentials at the electrode locations by

(1) W=L∗S,

where *L* is the lead-field matrix, *S* is the sources, and *W* is the measurements.

### 2.3. Electrode to Source Signal (Inverse) Modeling

Inverse modeling is the most important process, since improved classification performance using the cortical sources computed from electrode measurements is our goal. Since the sources outnumber the electrodes, we must set constraints and assume a head model or source to solve this problem. The scalp EEG signals are converted to cortical sources using the Brainstorm software from The Biomedical Imaging Group of the University of Southern California [15] using Standardized low-resolution brain electromagnetic tomography (sLORETA) [16]. This method calculates the noise-normalized solution.

### 2.4. Region of Interest (ROI) Selection for Motor Imagery

**Figure 3:**
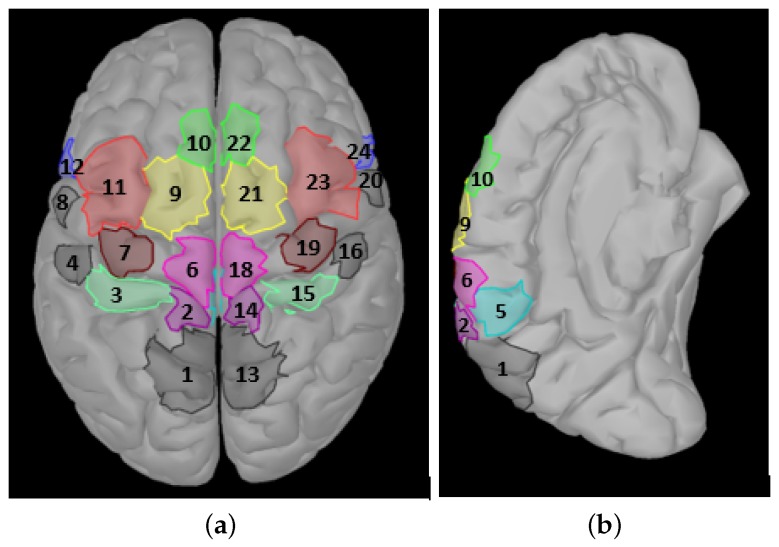
Selected brain regions: (**a**) top view, (**b**) midline surface.

The brain has cortical areas responsible for specific tasks. The subjects performed motor imagery tasks during their recordings in the dataset used for training and tests. The somatosensory cortical areas responsible for the tasks to be classified are included in the feature vectors (as in [10]) to improve classification performance. Region of interest areas are shown in Figure 3.

1. somatosensory association cortex (SAC),
2. primary foot somatosensory area (S1F),
3. primary hand somatosensory area (S1H),
4. secondary somatosensory area (S2),
5. cingulate motor area (CMA),
6. primary foot motor area (M1F),
7. primary hand motor area (M1H),
8. primary lip motor area (M1L),
9. supplementary motor area (SMA),
10. presupplementary motor area (pSMA),
11. dorsal premotor cortex (PMd),
12. ventral premotor cortex (PMv) areas.

The analysis was performed inside these regions, except for the first, fourth, and eighth regions because these ROIs are not related to programming.

### 2.5. CSP Features

Extracting meaningful information from raw data to reduce the dimension or number of inputs to a classifier is called feature extraction. Features represent raw data and should not include redundant information. The raw data in this paper comprise EEG signals recorded using 118 electrodes on the scalp. Common spatial pattern (CSP) features are extracted both from the recorded EEG and from the computed cortical signals.

The most commonly used method to classify EEG signals, such as those observed during motor imagery, is the CSP. These are optimal spatial filters that maximize the differences of variance between two classes [17]. In the literature, CSP features have been successfully applied to EEG signal classification for two-class problems [18]. This method uses class labels (in this study, right hand and right foot) and forms spatial filters so that the variance of the filtered data of one class is maximized, while the variance of the filtered data from the other classes is minimized [19]. Thus, the resulting feature vectors enhance the discriminability between two classes. CSP feature vectors are given to an SVM classifier to train and then tested to evaluate their performance.

## 3. Results

Most of the studies reported in the literature focused on sensor-based BCIs. First, raw sensor data are filtered into two groups in this study: 0.1–30 Hz and μ rhythm (8–13 Hz).

Table 2 lists the classification success of sensor data for 118 channels. Classification performance is determined by true/false predictions for each case to determine the success of the predictions, which is calculated by

(2) $$\mathrm{Classification}\mathrm{performance}=\frac{\mathrm{Number}\mathrm{of}\mathrm{correct}\mathrm{predictions}}{\mathrm{Total}\mathrm{number}\mathrm{of}\mathrm{predictions}}*100$$

The sensor data analysis resulted in a success ratio of 59.82%, 87.50%, 60.71%, 54.46%, and 51.59% for recordings from five subjects using a 0.1–30 Hz frequency range. Even though the performance was satisfactory, some imagery motor commands of the subjects were misclassified. Since the μ rhythm (8–13 Hz) is related to motor activities, the successful performance of the μ rhythm signals was 69.64%, 100%, 71.43%, 76.34%, and 50.4%.

In this study, cortical sources were calculated using the sLORETA inverse problem solution. Then, a classification of the same dataset was performed using neural sources transformed from scalp EEG electrodes. The analysis is categorized into three main groups: a left lobe analysis, a right lobe analysis, and an analysis of the combined (left + right) lobes to understand the motor imagery activations in the brain and better understand which regions of the brain have more information about the right hand or right foot motor imagery. These analyses were carried out for both the 0.1–30 Hz and μ frequency bands. The results are computed using a subset of nine areas, including (S1F), (S1H), (CMA), (M1F), (M1H), (SMA), (pSMA), (PMd), and (PMv). The left lobe results are calculated $2^{9}$ times for all possible subsets and run with a number of CSP filter pairs.

The best results are given for the sources in the selected set of ROIs in Table 3. The “left lobe max” column presents the best results achieved from all subsets of the sources in nine ROIs ($2^{9}$ times) using different CSP filter pair parameters. The third column lists the best results for subsets of three elements in the left lobe, the fourth column lists the best results for all possible subsets of the right lobe, the fifth column lists the best results for subsets of the three elements in the right lobe, and the sixth column lists the best results for the subsets of six elements of all 18 ROIs from both the right and left lobes using six pairs of CSP filters.

Sensitivity (sens) and specificity (spec) measures are also calculated. The right hand is selected as a positive case and the right foot as a negative case for the calculation of the sensitivity and specificity values.

(3) $$\mathrm{sensitivity}=\frac{\mathrm{Number}\mathrm{of}\mathrm{true}\mathrm{positives}}{\mathrm{Number}\mathrm{of}\mathrm{true}\mathrm{positives}+\mathrm{Number}\mathrm{of}\mathrm{false}\mathrm{negatives}}=\frac{\mathrm{TP}}{\mathrm{TP}+\mathrm{FN}}$$

(4) $$\mathrm{specificity}=\frac{\mathrm{Number}\mathrm{of}\mathrm{true}\mathrm{negatives}}{\mathrm{Number}\mathrm{of}\mathrm{true}\mathrm{negatives}+\mathrm{Number}\mathrm{of}\mathrm{false}\mathrm{positives}}=\frac{\mathrm{TN}}{\mathrm{TN}+\mathrm{FP}}$$

The ROIs in the left lobe has a higher overall classification accuracy, as shown in Table 3. A slight classification degradation of 4% is observed using only 3 ROIs compared to all the ROI combinations from a single lobe. A maximum classification accuracy of 75.5% was achieved using combinations of six ROIs from the two lobes on average. This result is 7% higher than that of the 3 ROIs from the left lobe and 9% higher than that of the 3 ROIs from the right lobe. Also, this result was 3% and 4% better than that of the left and right lobes’ maximum classification accuracy, respectively. Subject al has the best imagery performance (98.21%), whereas subject av has the worst, with only 64.80%, as seen in the table. Average accuracy in six regions from both lobes are higher than that of the accuracy of all other combinations. Subject al achieved maximum sensitivity and specificity. Although sensitivities are lower than accuracy in some local regions, specificities are higher. Similarly, sensitivities are high for low specificities.

In Table 4, the most successful areas are given in the related Table 3 results.

The regions corresponding to the best classification results of Table 3 are given in Table 4 as the contributing ROIs at the cortical level. Unfortunately, the regions are not the same for different subjects. The first column of Table 4 indicates that the $S1H_{L}$ and $M1F_{L}$ regions appeared five times, and $CMA_{L}$ and $M1H_{L}$ regions appeared four times, in the set of regions corresponding to the best results. The second column indicates that $CMA_{L}$ and $SMA_{L}$ appear three times in the three-region sets corresponding to the best results.

On the other hand, the right lobe results are more interesting, as the $CMA_{R}$ region was observed five times, and the $M1F_{R}$ region appeared four times, in the best results. This shows that the right lobe can be as active as the left lobe, which was unexpected. Lastly, the ROIs at the cortical level of six regions from both lobes are listed in the last column corresponding to the best results. The $S1F_{L}$ and $S1F_{R}$ regions provide the best results for the 0.1–30 Hz filtered data.

**Figure 4:**
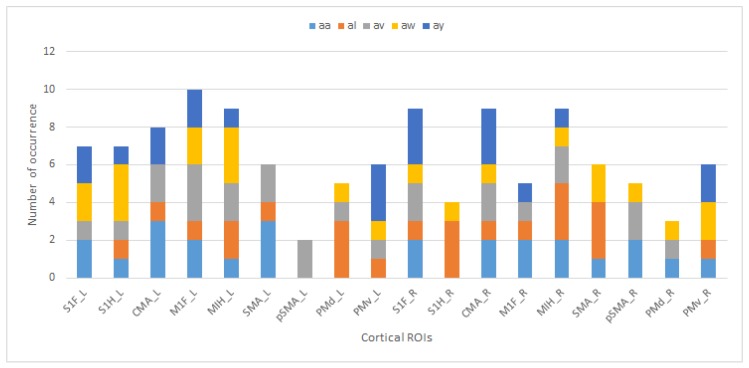
The 0.1–30 Hz ROI occurrence chart: primary foot somatosensory area (S1F), primary hand somatosensory area (S1H), cingulate motor area (CMA), primary foot motor area (M1F), primary hand motor area (M1H), supplementary motor area (SMA), presupplementary motor area (pSMA), dorsal premotor cortex (PMd), ventral premotor cortex (PMv), _L indicates the left lobe, _R indicates the right lobe.

The regions and their occurrence numbers from Table 4 for each subject with unique colors are shown to indicate the subjects’ and regions’ contributions to the best results in Figure 4.

The μ band results are given in Table 5. The “Left lobe max” column presents the best results achieved from all subsets of the sources in the nine ROIs ($2^{9}$ times) using different CSP filter pair parameters. The third column lists the best results for subsets of the three elements in the left lobe, the fourth column lists the best results for all possible subsets of the right lobe, the fifth column lists the best results for subsets of the three elements in the right lobe, and the sixth column lists the best results for subsets of the six elements of all 18 ROIs from both the right and left lobes using six pairs of CSP filters.

The μ band analysis results are given in Table 5. Filtering signals out of the μ band improved the classification accuracy, sensitivity and specificity compared to the 0.1–30 Hz band signal results, which supports Table 3. The left lobe accuracy is better than the right lobe accuracy, as seen in Table 5. Here, a slight classification degradation of 3% and 7.5% can be achieved using only three ROIs in the left lobe and right lobe, respectively, compared to the best average results of all ROI combinations for one lobe. The maximum classification accuracy can be achieved using ROIs from two lobes. This accuracy is 5% and 13% better than that using only three ROIs from the left and right lobes. This accuracy is also 2% and 5% better than that of the best results from all ROI combinations from the left and right lobes. The subject-based results are similar to those in Table 3, as the subject al has the best imagery performance at 100% classification accuracy, and subject av has the worst performance at only 67.86%.

Best sensitivity and specificity are achieved in six regions from both lobes. Sensitivity and specificity achieved from the left lobe is better than that of those from the right lobe. Subject al achieved 100% sensitivity and specificity whereas Subject av achieved 69.23% sensitivity and 66.67% specificity.

In Table 6, the most successful areas are given in the related Table 5 results.

The left lobe μ band analysis indicates that the $S1H_{L}$ and $SMA_{L}$ regions appeared for all subjects, but the $M1F_{L}$ and $M1H_{L}$ regions appeared three times in the best results. For the three-region combinations of subsets, the $S1H_{L}$ region was observed three times in the best results.

The right lobe μ band analysis indicates the $pSMA_{R}$ region five times and the $S1F_{R}$, $S1H_{R}$, $M1H_{R}$ and $PMd_{R}$ regions four times in the best results. For the three-region combinations of subsets, $M1H_{R}$, $SMA_{R}$ and $pSMA_{R}$ appear three times in the best results. The best classification results for all tests were achieved using both the right and left lobes in the μ band. The $S1H_{L}$ and $M1H_{L}$ regions appeared four times, but $SMA_{R}$ and $PMd_{R}$ appeared three times in the best results.

**Figure 5:**
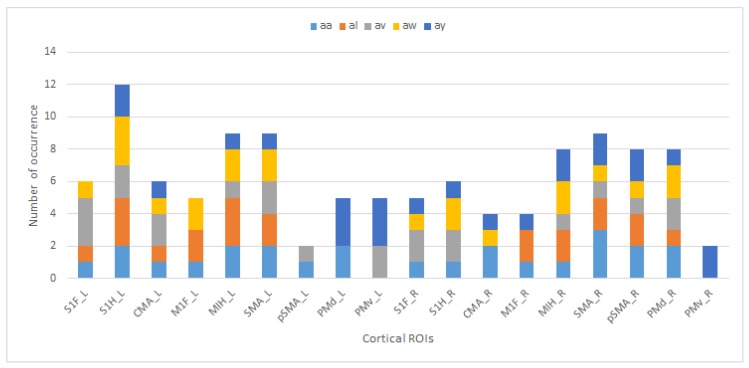
μ band ROI occurrence chart: Primary foot somatosensory area (S1F), primary hand somatosensory area (S1H), cingulate motor area (CMA), primary foot motor area (M1F), primary hand motor area (M1H), supplementary motor area (SMA), presupplementary motor area (pSMA), dorsal premotor cortex (PMd), ventral premotor cortex (PMv). _L indicates left lobe, _R indicates right lobe.

The regions and their occurrence numbers from Table 6 for each subject with unique colors are shown to indicate the subjects’ and regions’ contributions to the best results in Figure 5.

## 4. Discussion

This study aimed to find the most relevant cortical regions of the brain responsible for motor imagery of the right foot and right hand. EEG electrode signals were first transformed into cortical source signals by an inverse solution, and an SVM classifier was then trained to classify all subsets of the cortical sources to find those that contribute the most to the motor imagery of the right hand and right foot. In the tables, the most accurate results and related regions are given. The classification accuracies listed in the Tables revealed that the accuracy varies for each group and region, so the best accuracy is different for each subject. Also, the band-pass frequency of the signals in 0.1–30 Hz delta, theta, alpha, and beta rhythms and 8–13 Hz μ rhythms were used to compare and determine the most relevant regions and achieve success for each group.

The left lobe analysis for the 0.1–30 Hz band provided better results than the right lobe. Competition database maintainers replied to our request to supply hand preferences of subjects. They are all right-handed except Subject al. Foot preferences of the subjects are not specified in the dataset. The dominant hemisphere may have a better result depending on one’s preference for the right or left extremity. Since there is only one left-handed Subject in the dataset, the comparison may not be accurate. The regions $M1F_{L}$, $M1H_{L}$, $S1F_{R}$, $CMA_{R}$ and $M1H_{R}$ in the 0.1–30 Hz band and $S1H_{L}$, $M1H_{L}$, $SMA_{L}$ and $SMA_{R}$ in the μ band were the most dominant among the results. The S1F, S1H, M1H, and M1F regions were observed in the most successful classification results. Our findings show that these regions are related to motor imagery. In 2011, Pelgrims et al. [20] studied the primary motor cortex’s contribution to motor imagery and showed the relationship between these tasks. In 2006, Neuper et al. [21] stated that preparation, execution, and imagination activate sensorimotor areas in the μ band.

The $SMA_{L}$ region is among the most successful regions in μ band analysis. This is in good agreement with the study published in 2015 by Dalla–Corte [22], which observed SMA activity while performing right and left-handed motor tasks. This result was also confirmed by an fMRI analysis of these regions. SMA activity was also observed during motor imagery tasks in the same study. The SMA and pSMA regions have a relationship with motor function planning, processing, and execution [23,24].

CMA is one of the most successful regions in 0.1–30 Hz band analysis, as shown in Figure 4. The study of Athanasiou et al. [25] presented an analysis of the motor execution of α and β band connectivity. Their findings show that CMA has connections with premotor areas. The S1H region is common for all subjects in μ band analysis for the left lobe. The $M1F_{L}$ and $M1H_{L}$ regions are activated in subject al and subject aw where the accuracy is higher compared to subject aa and subject av, in both the current study and in the literature. More interestingly, the accuracy does not drop below 87% when regions $M1F_{L}$ and $M1H_{L}$ appear at the same time. Most of the time, the left hemisphere gives more accurate results, as expected. It is known that right-handed or right-armed people use the left hemisphere. In μ rhythm activations, the left lobe is more successful than the right lobe, and in the 0.1–30 Hz band, the Subject av’s right lobe gives better results than the left lobe.

In the literature and the BCI Competition III Dataset IVa results page, we see that Subject aa and Subject av’s success is lower, despite having more training data compared to Subject aw and Subject ay. Subject ay has only 28 training data, and its accuracy is higher. In addition to the cortex, subcortical areas also contribute to the programming of motor function for classic information. There is no direct record of the EEG data for subcortical fields. In this study, the primary motor areas, as well as the premotor areas and supplementary areas, were included in the selection of the cortex areas. Selecting the areas of the primary motor areas $M1F_{L}$ and $M1H_{L}$ increased the accuracy of the subjects (subjects who could increase their activity in areas with $M1F_{L}$ and $M1H_{L}$ motor movements achieved higher accuracy).

Lastly, we analyzed two lobes. For better analysis, we restricted the study to 6 regions to determine the most important areas. This was done to determine if using only the left areas could achieve better accuracy than using both lobes. The classification accuracy was increased compared to single lobe calculations. In this analysis, four occurrences of the regions $S1F_{L}$ and $S1F_{R}$ are in the best results, and also four occurrences of $S1H_{L}$ and $M1H_{L}$ are in the best results of μ band analysis which ensure commonality between the subjects.

It was surprising that the results for every subject using the left and right lobe together were better compared to those using left lobes only. In 2004, Hoshi and Tanji [26] stated that the PMd and PMv areas are related to planning and execution. The successful regions found in our work based on differentiating signals are similar to the areas in this previous study. A left lobe of 0.1–30 Hz $S1H_{L}$ and $M1F_{L}$, for the μ band region $S1H_{L}$ most commonly appeared in the successful results. The right lobe $CMA_{R}$ appeared more at 0.1–30 Hz and $pSMA_{R}$ for the μ band. The best results were obtained using two lobes. The $S1F_{L}$ and $S1F_{R}$ pair had the most successful ROIs in 0.1-30 Hz and for the μ band $S1H_{L}$-$M1H_{L}$, and $SMA_{R}$-$PMd_{R}$ gave the best results.

Motor imagery EEG signals from a dataset are classified at the sensor level and the cortical level. Special attention is given to the comparison of the classification accuracy results of the cortical source level and the corresponding ROIs in this manuscript. Using cortical sources to classify motor imagery improves performance. The cortical sources in some regions can be used for successful BCI, even though the inverse problem must be solved to compute the cortical sources. The recorded right hand and right foot motor imagery EEG signals were used and successfully classified. The source space classification performance is promising, even though source signal computation is based on an inverse problem solution. The classification accuracy of motor imagery based BCI was improved using source space. The neurophysiological constraints while processing data not only improved the calculation time but also increased classification success. Using the source space via an inverse problem solution and biophysical knowledge to select the regions associated with motor functions improved the classification accuracy of the BCI technology.

There are many parameters, such as filter, head model, and source assumptions, that impact the inverse solution for computing cortical sources while processing EEG signals. Invasive BCIs are not applicable because many situations that threaten human health can occur during such operations. Semi-invasive BCIs can be used for some applications that control basic devices but can cause infections. Non-invasive BCIs based on EEG recordings do not pose any risk, and research has lately been focused on improving the performance of BCIs. Successful BCIs have been designed and built by teams over the last few decades. Even though the results given in this manuscript are promising, there are some limitations to this analysis. The dataset, for example, contained EEGs recorded from only five healthy subjects.

Future research should increase the number of subjects and trials and use real patients’ data (e.g., amputees or paralyzed patients) to confirm the findings reported here. Another limitation is that the real MRIs of subjects are not given. A template head model was used for the subjects. It is known that the cortical regions can be expanded or become narrower depending on the dominant usage of specific regions because the brain possesses neural plasticity. Therefore, interdisciplinary studies in the neurophysiology and neuroengineering fields could provide a significantly better understanding of brain activities.

## 5. Conclusions

Accurate BCI design remains an active research area. Developments in both hardware and software help designers to improve BCIs beyond imagination. Future BCIs are expected to have better classification accuracy compared to the BCIs of today and will be used in daily life. Improved classification performance is possible using information from Physiology to focus on cortical sources in relevant brain regions. Advances in BCI design will certainly improve the quality of the lives of amputees and paralyzed people and let them interact successfully with their environment. Our findings showed that the activity of the cortical regions can vary slightly among subjects. Using both $M1F_{L}$ and M1H regions in a 0.1–30 Hz range and using M1H, $S1H_{L}$, and SMA regions in μ band signals achieved better classification accuracy. For a successful BCI, one needs to choose cortical regions carefully in order to include relevant regions and not to include regions that degrade classification.

## Figures and Tables

**Table 1:** Subjects in the dataset and the number of training and testing signals.

	Number of Training Samples	Number of Test Samples
aa	168	112
al	224	56
av	84	196
aw	56	224
ay	28	252

**Table 2:** Sensor level classification accuracies.

	Sensor Data 0.1–30 Hz (118 Electrodes)	μ Band (118 Electrodes)
aa	59.82	69.64
al	87.50	100
av	60.71	71.43
aw	54.46	76.34
ay	51.59	50.40

**Table 3:** Accuracy (acc), sensitivity (sens) and specificity (spec) of all combinations for 0.1–30 Hz range.

0.1–30 Hz	Criteria	Left Lobe Max	Left Lobe 3 Regions	Right Lobe Max	Right Lobe 3 Regions	Left and Right Lobe 6 Regions
aa	acc	67.86	63.39	66.96	63.39	68.75
	sens	67.65	75.68	79.49	70.21	79.07
	spec	68.18	57.33	60.27	58.46	62.32
al	acc	92.86	85.71	89.29	83.93	98.21
	sens	100	85.71	89.29	91.30	100
	spec	87.50	85.71	89.29	78.79	96.55
av	acc	61.73	58.67	64.80	62.24	64.80
	sens	60.55	57.94	67.06	59.23	63.81
	spec	63.22	59.55	63.06	68.18	65.93
aw	acc	78.57	75.45	75.00	68.30	79.46
	sens	75.41	69.78	74.55	64.03	82.00
	spec	82.35	84.71	75.44	75.29	77.42
ay	acc	60.71	57.94	59.92	55.95	66.27
	sens	56.42	54.71	55.07	52.56	60.11
	spec	71.23	64.63	82.22	75.68	82.61
Average	acc	72.35	68.23	71.19	66.76	75.50
	sens	72.01	68.76	73.09	64.47	77.00
	spec	74.50	70.39	74.06	71.28	76.97

**Table 4:** The Most successful ROIs for 0.1–30 Hz signals: primary foot somatosensory area (S1F), primary hand somatosensory area (S1H), cingulate motor area (CMA), primary foot motor area (M1F), primary hand motor area (M1H), supplementary motor area (SMA), presupplementary motor area (pSMA), dorsal premotor cortex (PMd), ventral premotor cortex (PMv). Subscript L indicates left lobe, Subscript R indicates right lobe.

0.1–30 Hz	Left Lobe Max	Left Lobe 3 Regions	Right Lobe Max	Right Lobe 3 Regions	Left and Right Lobe 6 Regions
aa	$S1F_{L},$ $S1H_{L},$ $CMA_{L},$ $M1F_{L},$ $M1H_{L},$ $SMA_{L}$	$CMA_{L},$ $M1F_{L},$ $SMA_{L}$	$S1F_{R},$ $CMA_{R},$ $M1F_{R},$ $SMA_{R},$ $pSMA_{R},$ $PMd_{R},$ $PMv_{R}$	$CMA_{R},$ $M1F_{R},$ $M1H_{R}$	$S1F_{L},$ $CMA_{L},$ $SMA_{L},$ $S1F_{R},$ $M1H_{R},$ $pSMA_{R}$
al	$S1H_{L},$ $CMA_{L},$ $M1F_{L},$ $PMd_{L},$ $PMv_{L}$	$M1H_{L},$ $SMA_{L},$ $PMd_{L}$	$S1H_{R},$ $CMA_{R},$ $M1F_{R},$ $M1H_{R},$ $SMA_{R},$ $PMv_{R}$	$S1H_{R},$ $M1H_{R},$ $SMA_{R}$	$M1H_{L},$ $PMd_{L},$ $S1F_{R},$ $S1H_{R},$ $M1H_{R},$ $SMA_{R}$
av	$S1H_{L},$ $CMA_{L},$ $M1F_{L},$ $M1H_{L},$ $SMA_{L},$ $pSMA_{L},$ $PMd_{L},$ $PMv_{L}$	$CMA_{L},$ $M1F_{L},$ $SMA_{L}$	$CMA_{R},$ $M1F_{R},$ $M1H_{R},$ $pSMA_{R},$ $PMd_{R}$	$S1F_{R},$ $M1H_{R},$ $pSMA_{R}$	$S1F_{L},$ $M1F_{L},$ $M1H_{L},$ $pSMA_{L},$ $S1F_{R},$ $CMA_{R}$
aw	$S1F_{L},$ $S1H_{L},$ $M1F_{L},$ $M1H_{L},$ $PMd_{L}$	$S1H_{L},$ $M1H_{L},$ $PMv_{L}$	$S1F_{R},$ $S1H_{R},$ $CMA_{R},$ $M1H_{R},$ $PMd_{R}$	$SMA_{R},$ $pSMA_{R},$ $PMv_{R}$	$S1F_{L},$ $S1H_{L},$ $M1F_{L},$ $M1H_{L},$ $SMA_{R},$ $PMv_{R}$
ay	$S1H_{L},$ $CMA_{L},$ $M1F_{L},$ $M1H_{L},$ $PMv_{L}$	$S1F_{L},$ $CMA_{L},$ $PMv_{L}$	$S1F_{R},$ $CMA_{R},$ $M1F_{R},$ $PMv_{R}$	$S1F_{R},$ $CMA_{R},$ $PMv_{R}$	$S1F_{L},$ $M1F_{L},$ $PMv_{L},$ $S1F_{R},$ $CMA_{R},$ $M1H_{R}$

**Table 5:** Accuracy (acc), sensitivity (sens) and specificity (spec) of all combinations for μ band signals.

8–13 Hz	Criteria	Left Lobe Max	Left Lobe 3 Regions	Right Lobe Max	Right Lobe 3 Regions	Left and Right Lobe 6 Regions
aa	acc	66.96	62.50	69.64	61.61	74.11
	sens	69.49	65.52	74.07	71.79	80.39
	spec	64.15	59.26	65.52	56.16	68.85
al	acc	100	98.21	94.64	91.07	100
	sens	100	96.55	90.32	87.10	100
	spec	100	100	100	96.00	100
av	acc	66.84	60.71	66.33	62.24	67.86
	sens	65.71	60.40	67.39	63.33	69.23
	spec	68.13	61.05	65.38	61.32	66.67
aw	acc	87.05	84.38	86.16	80.80	90.18
	sens	85.84	83.78	89.11	81.90	90.74
	spec	88.29	84.96	83.74	79.83	89.66
ay	acc	86.11	86.11	75.00	58.73	86.11
	sens	81.29	81.29	67.05	55.06	80.85
	spec	92.04	92.04	92.41	67.57	92.79
Average	acc	81.39	78.38	78.35	70.89	83.65
	sens	80.47	77.51	77.59	71.84	84.24
	spec	82.52	79.46	81.41	72.18	83.59

**Table 6:** Most successful ROIs for μ band signals: primary foot somatosensory area (S1F), primary hand somatosensory area (S1H), cingulate motor area (CMA), primary foot motor area (M1F), primary hand motor area (M1H), supplementary motor area (SMA), presupplementary motor area (pSMA), dorsal premotor cortex (PMd), ventral premotor cortex (PMv). Subscript L indicates left lobe, subscript R indicates right lobe.

8–13 Hz	Left Lobe Max	Left Lobe 3 Regions	Right Lobe Max	Right Lobe 3 Regions	Left and Right Lobe 6 Regions
aa	$S1F_{L},$ $S1H_{L},$ $M1F_{L},$ $SMA_{L},$ $pSMA_{L}$	$CMA_{L},$ $M1H_{L},$ $PMd_{L}$	$S1F_{R},$ $S1H_{R},$ $CMA_{R},$ $M1F_{R},$ $M1H_{R},$ $SMA_{R},$ $pSMA_{R},$ $PMd_{R},$	$CMA_{R},$ $SMA_{R}$ $pSMA_{R}$	$S1H_{L},$ $M1H_{L},$ $SMA_{L},$ $PMd_{L},$ $SMA_{R},$ $PMd_{R}$
al	$S1H_{L},$ $M1F_{L},$ $M1H_{L},$ $SMA_{L},$	$S1H_{L},$ $M1H_{L},$ $SMA_{L},$	$M1F_{R},$ $M1H_{R},$ $SMA_{R},$ $pSMA_{R},$ $PMd_{R},$	$M1H_{R},$ $SMA_{R},$ $pSMA_{R}$	$S1F_{L},$ $S1H_{L},$ $CMA_{L},$ $M1F_{L},$ $M1H_{L},$ $M1F_{R},$
av	$S1F_{L},$ $S1H_{L},$ $CMA_{L},$ $M1H_{L},$ $SMA_{L},$ $PMv_{L},$	$S1F_{L},$ $pSMA_{L},$ $PMv_{L}$	$S1F_{R},$ $S1H_{R},$ $pSMA_{R},$ $PMd_{R}$	$S1F_{R},$ $S1H_{R},$ $PMd_{R}$	$S1F_{L},$ $S1H_{L},$ $CMA_{L},$ $SMA_{L},$ $M1H_{R},$ $SMA_{R}$
aw	$S1H_{L},$ $M1F_{L},$ $M1H_{L},$ $SMA_{L}$	$S1F_{L},$ $S1H_{L},$ $CMA_{L},$	$S1F_{R},$ $S1H_{R},$ $M1H_{R},$ $pSMA_{R},$ $PMd_{R},$	$S1H_{R},$ $CMA_{R},$ $M1H_{R}$	$S1H_{L},$ $M1F_{L},$ $M1H_{L},$ $SMA_{L},$ $SMA_{R},$ $PMd_{R},$
ay	$S1H_{L},$ $SMA_{L},$ $PMd_{L},$ $PMv_{L}$	$S1H_{L},$ $PMd_{L},$ $PMv_{L}$	$S1F_{R},$ $S1H_{R},$ $CMA_{R},$ $M1F_{R},$ $M1H_{R},$ $SMA_{R},$ $pSMA_{R},$ $PMv_{R}$	$M1H_{R},$ $SMA_{R},$ $pSMA_{R}$	$CMA_{L},$ $M1H_{L},$ $PMd_{L},$ $PMv_{L},$ $PMd_{R},$ $PMv_{R}$

## Abbreviations

The following abbreviations are used in this manuscript:

BCI	Brain–computer interface
EEG	Electroencephalogram
sLORETA	Standardized low-resolution brain electromagnetic tomography
CSP	Common spatial patterns
SVM	Support vector machine
MEG	Magnetoencephalography
fMRI	Functional Magnetic Resonance Imaging
ROI	Region of Interest
SAC	somatosensory association cortex
S1F	primary foot somatosensory area
S1H	primary hand somatosensory area
S2	secondary somatosensory area
CMA	cingulate motor area
M1F	primary foot motor area
M1H	primary hand motor area
M1L	primary lip motor area
SMA	supplementary motor area
pSMA	presupplementary motor area
PMd	dorsal premotor cortex
PMv	ventral premotor cortex
TP	True positive
TN	True negative
FP	False positive
FN	False negative
acc	accuracy
sens	sensitivity
spec	specificity
